# The Effect of Various Si/Al, Na/Al Molar Ratios and Free Water on Micromorphology and Macro-Strength of Metakaolin-Based Geopolymer

**DOI:** 10.3390/ma14143845

**Published:** 2021-07-09

**Authors:** Hongguang Wang, Hao Wu, Zhiqiang Xing, Rui Wang, Shoushuai Dai

**Affiliations:** 1 School of Civil Engineering, Northeast Forestry University, Harbin 150040, China; hao@nefu.edu.cn (H.W.); xingzhiqiang_nefu613@126.com (Z.X.); wangrui20075039@126.com (R.W.); daishoushuai@nefu.edu.cn (S.D.); 2 Key Laboratory of Bio-Based Material Science and Technology (Ministry of Education), Northeast Forestry University, Harbin 150040, China

**Keywords:** metakaolin-based geopolymers, Si/Al, Na/Al molar ratio, morphology, free water, mechanical properties

## Abstract

The current work aimed to explore the effect of Na/Al ratios of 0.43, 0.53, 0.63, 0.73, 0.83, and 0.93, using NaOH to alter the molar ratio, on the mechanical properties of a geopolymer material, with fixing of the Si/Al molar ratio. While fixing the Na/Al molar ratio, alteration of the Si/Al ratios to 1.7, 1.75, 1.8, 1.85, 1.9, 1.95 was used, with silica fume and sodium silicate as a silica corrector. The influence on the micromorphology and macro-strength of samples was characterized through SEM, EDS, and compressive strength characterization methods. The results show that Si/Al and Na/Al molar ratios play a significant role in the microstructure and mechanical behavior of MK-based geopolymers, and revealed that the optimal molar Si/Al and Na/Al ratios for attaining maximum mechanical strength in geopolymers are 1.9 and 0.73, respectively. Under various Si/Al ratios, the macro-strength of the geopolymer mainly relies on the formation of NASH gel, rather than zeolites or silicate derivatives. The appropriate Na/Al molar ratio can contribute to the geopolymerization, but a ultra-high Na/Al molar ratio caused a high alkali state that destroyed the microstructure of the geopolymers. Regardless of the amount of water contained in the initial geopolymer raw material, the water content of Si/Al = 1.65 and Si/Al = 1.75 after curing for 10 days was almost the same, and the bound water content of the final geopolymer was maintained at about 15%. Structural water exists in geological polymer gels in the form of a chemical structure. It has effects on the structural performance strength, while free water affects the volume stability of the geological polymer. Overall, the current work provides a perspective on the elemental composition analysis, combined with the molecular structure and micromorphology, to explore the mechanical performance of geopolymers.

## 1. Introduction

**Figure 1:**
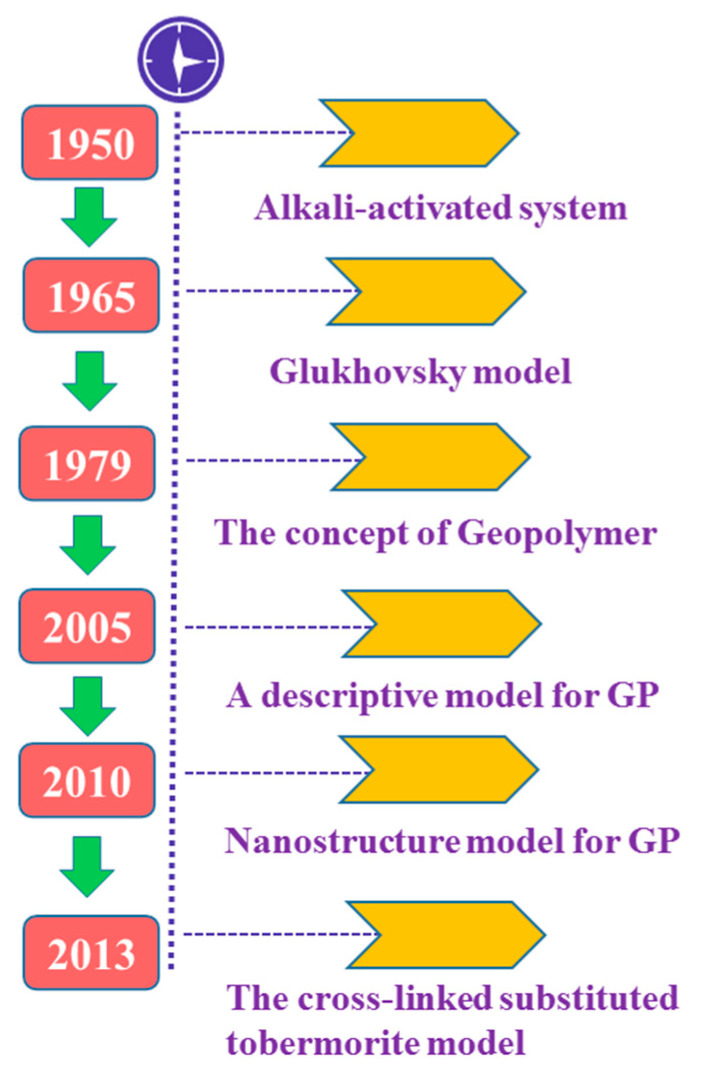
Development history of geopolymers.

In the 1940s, the Belgian scientist Purdon [1] found that ground blast furnace slag mixed with NaOH solution created the phenomenon of coagulation and hardening during experiments. Based on this, he put forward the “alkali excitation” theory for the first time and initiated the prelude to geopolymer research. In the 1950s, former Soviet Union scholar Glukhovsky [2] and others further studied the alkali activated characteristics of blast furnace slag, proposed the concept of “alkali activated cementitious material”, and established the classical “Glukhovsky” polymerization model. Prof. Joseph Davidovits in the 1970s made a systematic investigation of raw materials, the polymerization principle, and internal structure of geopolymers, and formed the concepts of “mineral polymer” [3] and “geopolymer” [4]. This presented the road map for the subsequent years of skyon geopolymer science innovation and research. Palomo et al. [5], in the year of 2005, studied and observed the changes of phase microstructure of fly ash at different stages of alkali excitation reaction by means of SEM and TEM, and proposed a more detailed descriptive reaction mechanism model at a microscopic level, based on the Glukhovsky model. In 2010, Criado [6] proposed a nanostructure geopolymer model. Subsequently, in 2013, Myers et al. [7], based on the SGM model [8] proposed by Richardson and Groves, discovered that hydrated calcium aluminosilicate (sodium) gel (C-(N)-A-S-H) can be significantly cross-linked by the study of alkali-activated slag in the laboratory, and proposed the cross-linked substituted tobermorite model (CSTM). The CSTM can better describe the spectrum and density information of materials, distinguish the proportion of crosslinking and non-crosslinking in the gel, and calculate the average chain length of C-(N)-A-S-H gel. The specific representative time course is shown in Figure 1.

Geopolymers are a kind of inorganic cementitious material with a three-dimensional network structure, formed by natural minerals or industrial wastes of active aluminosilicate materials as solid materials under high alkali conditions. Compared with traditional inorganic cementitious materials, such as Portland cement, a geopolymer has no hydration reaction with calcium silicate and mainly consists of ionic bonds and covalent bonds, which is similar to natural zeolite mineral. The geopolymer of each system showed excellent durability, especially the resistance to sulfate attack and acid attack [9,10], which was significantly better than traditional cement-based materials. Due to the excellent mechanical strength, fire resistance, corrosion resistance, low thermal conductivity, solidification of heavy metal ions, and energy conservation, its application was no longer limited to the construction field, but began to be used in high-performance composite materials [11], fireproof and high temperature-resistant materials [12], rapid repair concrete and subgrade aggregate materials [13,14], waterproof coating materials [15], the solidification of heavy metal waste [16], and aerospace and other fields [17].

**Figure 2:**
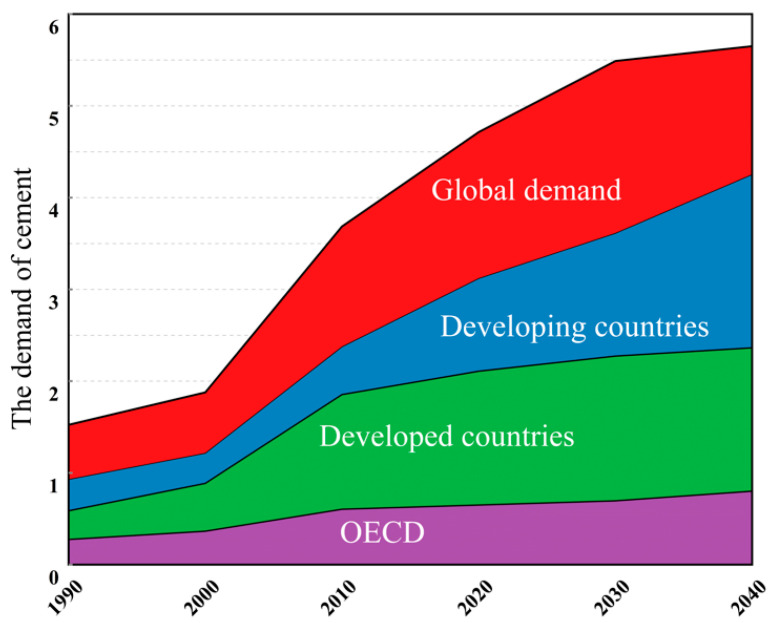
Global cement demand.

As can be seen from Figure 2, the growth in cement demand in diverse countries will increase steadily over the next 30 years or more. Although the rate of increase of demand for cement has slowed, it still keeps rising steadily, and the demand from developing countries is still very large. Worldwide, the production of Portland cement (PC) keeps increasing, by 9% per year. Due to the large amount of CO_2_ released into the atmosphere in the process of cement production, this increasing rate poses a great danger to the environment. The annual greenhouse gas emissions from PC production are about 1.5 billion tons, accounting for 6% of the average emissions of many industries in the world.

The production of Portland cement not only consumes a lot of resources and energy, it also brings serious environmental problems. Therefore, it is urgent to develop a new type of environmentally friendly green material to replace Portland cement. Geopolymer, as the most promising new green inorganic cementing material in the 21st century, has great potential to replace cement-based materials. Compared with cement, the energy consumption and waste gas emission of geopolymers are extremely low, and its energy consumption for production is 1/6~1/4 of cement, 1/70 of steel, 1/20 of ceramic, and 1/150 of plastic. The production process of geopolymers can be reduced by 60% for energy consumption and 80% for the emission of greenhouse gases, and it is regarded as “green concrete”.

From the perspective of the microstructure, geopolymers are composed of cross-linked silico-oxygen and aluminum-oxygen tetrahedral, with charge balancing basic cations such as Na+ or K+ ions. According to the Si/Al ratio, geopolymer structures can be divided into three types: silicon aluminum long chain PS type, double silicon aluminum long chain PSS type, and triple silicon aluminum long chain PSDS type. A geopolymer is an amorphous three-dimensional network polymer, formed by the condensation of silicon oxygen tetrahedrons and aluminum oxygen tetrahedrons. Its empirical structural formula is Mn (-(SiO_2_)_z_-Al_2_O_3_)n·wH_2_O, where M is alkali metal ions (such as Na^+^, K^+^, Ca^2+^); n is the degree of polymerization; z is the molar ratio of Si/Al; w is the content of bound water; and the symbol “-” indicates the chemical bond.

Recent studies have focused on evaluating the properties of formulations of granulated blast furnace slag [18,19], fly ash [20,21], and metakaolin-based geopolymers [22], showing the enormous potential of geopolymer development of an environmentally-friendly construction material. In this research, the emphasis was not the optimization of metakaolin-based geopolymer performance, but to explore the relationship between the macro-strength and micromorphology of samples and various Si/Al (Na/Al) molar ratios. Analyses of experimental results by SEM and EDS were conducted to present a systematic study of the development of the mechanical properties of geopolymers between 7 and 28 days with various Si/Al and Na/Al ratio compositions, to serve as a basis, correlated to their microstructure. In addition, to further illustrate the role of water during and after synthesis, metakaolin-based geopolymer samples with various Si/Al molar ratios were prepared.

## 2. Materials and Methods

### 2.1. Materials

(1) Metakaolin: metakaolin was chosen as the aluminosilicate material in this research due to its high purity, reactivity of components, and being more active for geopolymerization, compared with other materials such as fly ash, slag, etc. The metakaolin, made by calcining kaolin, was supplied by Jinao Refractory Materials Co., Ltd., China (Zhengzhou, China), and was used throughout this study. The general chemical composition of metakaolin powder is characterized in Table 1, using X-ray fluorescence (XRF) analysis and X-ray diffraction (XRD) experiment. The metakaolin was mainly composed of silica and alumina oxides, at the weight ratio of 1.36 (SiO_2_/Al_2_O_3_). The metakaolin used in this experiment was calcined at 650 °C to 850 °C low temperature with activity and physical properties as given in Table 2.

(2) Sodium silicate: water glass, otherwise known as sodium silicate, is a kind of water-soluble silicate. The molecular formula is Na_2_O·nSiO_2_, with the n representing the ratio of SiO_2_ to Na_2_O, called the water glass modulus, which is a very important parameter. The larger the n value, the higher the viscosity and strength. The different modulus of the exciter was adjusted by varying the amount of NaOH, and the sodium silicate solution needed to be set for 4 to 6 h to achieve equilibrium and to cool down to ambient temperature before the experiment was performed. The detailed mass compositions of sodium silicate are given in Table 3, as follows.

(3) NaOH: The analytical pure sodium hydroxide was sourced from Tanli Chemical Reagent Co., Ltd. (Tianjin, China) with a purity of 99%.
(4) Silica fume: Silica fume (SiO_2_ > 98%), purchased from Sichuan Langtian Resources Utilization Co., Ltd., China (Chengdu, China), was used as silica corrector to change Si/Al ratios. The particle size was measured at 50% of the cumulative under a size of 3 nm from SEM.

### 2.2. Experimental Preparation

#### 2.2.1. Mix Design

An experimental study was conducted for the purpose of evaluating the effects of various Na/Al and Si/Al molar ratios on the microstructure and mechanical properties of a metakaolin-based paste.

In order to study the effect of Si/Al ratio on the structure and properties of geopolymer, the Na/Al was fixed at 0.83 (if it is more than 0.83, there will be transient solidification), and the content of Si in the structure was adjust using silicon powder.

The aim was explore the influence of Na/Al ratio on the geopolymer; Si/Al = 1.85 was fixed, and the range of Na/Al was adjusted from 0.43 to 0.93 by adjusting the amount of NaOH. The specific mix design is illustrated in the Table 4 and Table 5 below.

#### 2.2.2. Experimental Procedure and Precautions

The metakaolin-based geopolymers were prepared from metakaolin, sodium silicate solution, alkali hydroxide, silica fume, and distilled water. Three group tests were carried out in this experiment. In the first group, while fixing the Na/Al ratio at 0.83, the Si/Al ratio varied from 1.7 to 1.95. The second group controlled the Si/Al ratio at 1.85, with the Na/Al ratio varying from 0.43 to 0.93. The third group explored the influence of water composition content. The Na/Al ratio was fixed at 0.83, and the Si/Al ratio was 1.65, 1.75, 1.85, and 1.95. The sealed and unsealed treatments were carried out at the early curing stage, and the changes of water molecules over 14 days were measured and recorded. All of the samples were cured at 60 °C for 6 h in an oven, and then cured at 25 °C at room temperature. The mechanical properties of the samples were tested for 14 days and 28 days.

**Figure 3:**
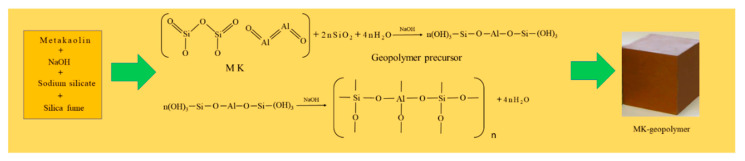
Diagram of geopolymer formation.

The experimental process of preparing metakaolin used in this study is described in the following four steps. The diagram of geopolymer formation is shown in Figure 3.

Step 1: In order to prepare a suitable alkali activator solution, the modulus of sodium silicate was adjusted by adding pre-weighed analytically pure NaOH particles to the industrial sodium silicate, to meet the needs of the experimental mix proportion. During the experiment, it was noteworthy that the NaOH particles released a lot of heat when dissolved in water, so the alkali activator solution needed to be stilled in advance to reach room temperature. Otherwise, the reaction process was intensified sharply due to the exothermic heat, resulting in the phenomenon of transient coagulation, the initial setting time was too short (less than 5 min), and it was impossible to complete the molding. However, the alkali activator solution should not be stilled for more than 6 h, or else condensation will occur due to too low a modulus. Therefore, it is recommend that the standing time should be 3 to 6 h.

Step 2: Weigh the amount of distilled water and metakaolin needed in the experiment, and fully and evenly mix with the pre-disposed alkali activated solution. Then put the prepared solution in the planetary cement mortar mixer, and stir slowly for 120 s and quickly for 120 s.

Step 3: After stirring, the fresh paste was then rapidly cast into 40 mm × 40 mm × 160 mm steel molds, while carrying out preliminary vibration, and then put on the vibration table for about 3 min to liberate the bubbles and be compacted.

Step 4: The samples were put into the laboratory oven at 60 °C for 6 h and an ambient temperature of 25 °C for 4 weeks. One group of samples were sealed with thin plastic at the exposed portion of the mold during the 6 h curing stage.

### 2.3. Characterization

#### 2.3.1. Unconfined Compressive Strength Test

In the experiment, a 4 cm × 4 cm × 4 cm cubic steel mold was used. Compressive strength was tested by using an electro-hydraulic servo machine with a capacity of 300 kN, and the loading rate was 2 mm/min. The strength was measured as the average of three duplicated samples according to the standard JGJ/T70-2009.

#### 2.3.2. Flexural Strength Test

The flexural strength test method of geopolymer mortar refers to the standard test method of cement sand loading. The prism specimen (4 cm × 4 cm × 16 cm) was loaded to failure by three-point bending loading. The bottom of the specimen had a two support spacing. The top loading point was located in the middle of the two support points and acted on the center of the specimen along the length direction. Record the load value at failure and calculate the bending strength according to the following formula.

(1) $$f_{b}=\frac{3Pl}{2b^{3}}$$

where *f_b_* is the flexural strength (MPa). *P* is the maximum failure load applied to the specimen in the middle of prism when broken (N). *l* is the spacing between the two fulcrums (mm). *b* is the side length of the square section of the prismatic specimen (mm).

The average value of three test blocks was taken as the flexural strength test result. When one of the three-point flexural strength values deviates 10% from the average value, the data is removed and the average value of the other values is taken as the result.

#### 2.3.3. Scanning Electron Microscopy and Energy Dispersive Spectrometer Analysis

The morphology of specimens at a microscale was observed with a scanning electron microscopy (SEM), with a Quanta 200 field emission scanning electron microscope, an acceleration voltage of 5 kV, and a maximum magnification of 1 million. Pieces with a size of 0.5 cm to 1 cm were selected from the crushed specimens and dried at 60 °C for 12 h. Before the morphology test, the sample was fixed on a metal carrier platform with conductive tape. Then, a gold spray was applied to the test samples for approximately 10 min, and conductive tape was used to connect the loading platform and the device base to increase the conductivity. The observation and freezing images were taken at relatively flat locations on the unpolished surface. During the observation, the local element composition was also characterized with an energy dispersive spectrometer (EDS).

#### 2.3.4. Calculation of Residual Water during Geopolymerization

In order to explore the changes of bound water and free water during the geopolymerization reaction, the initial water content in the raw material was measured, Na/Al = 0.83 and Si/Al = 1.65 to 1.95, respectively, and the mass changes of the experimental samples from 1 day to 14 days were recorded.

The percentage weight (*Wt_r_*) of residual water in the geopolymer was calculated using the equation as following.

(2) $$Wt_{r}\%=\frac{W_{H_{2}O}-{W^{\prime }}_{H_{2}O}}{W_{n}}\times 100\%$$

where *W*_H_2_O_, *W*′_H_2_O_, and *W_n_* were the initial weight of the water in the specimen, total water weight change of the specimen during curing and aging, and total weight of the specimen at the moment of measurement, respectively.

## 3. Results

### 3.1. Effect of Various Si/Al ratios on Microstructure and Mechanical Properties

In geopolymerization, the geopolymer formation is basically destructive, including dissolution and hydrolysis, followed by a coagulation process that occurs in an alkaline system; the concentrations of sodium and silicon determined the type of hydrolysis ion and the process of the condensation, showing prominent influence on the chemistry and physical properties of the geopolymer. Therefore, it is very important to investigate the effect of the Si/Al ratio on the micromorphology and mechanical behavior of MK-based geopolymers.

**Figure 4:**
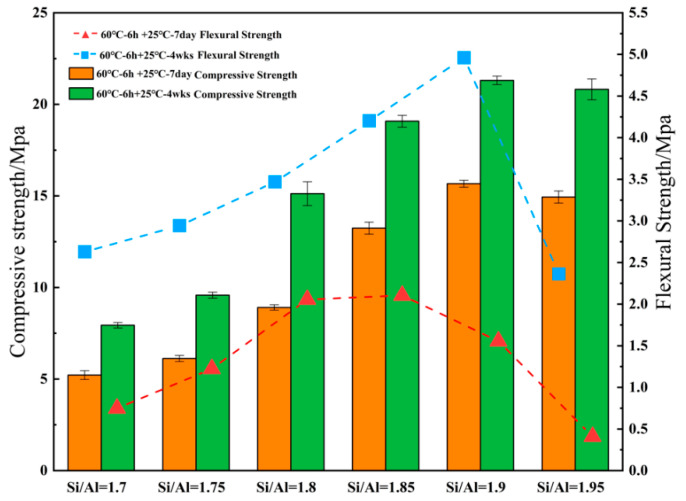
Compressive strength and flexural strength of geopolymer samples with various Si/Al ratios.

It can be seen from Figure 4 that the early strength of the geopolymer developed rapidly, and the strength basically reached more than 60% of its total strength in 7 days. This indicates that a geopolymer is a kind of early-strength cementitious material. When the Na/Al ratio was fixed at 0.83 and Si/Al = 1.7, the compressive strength at 7 days and 28 days was 5.2 MPa and 7.94 MPa, respectively. With increasing the Si/Al ratios, the strength of metakaolin increases and then decreases, and the strength reached the maximum of 21.3 MPa when the Si/Al ratio was 1.90. This is in agreement with the previous research results of other scholars. Steveson et al. [23] reported that geopolymers could obtain the best mechanical properties at an Si/Al ratio of the range of 1.50 to 1.95. Furthermore, De Silva et al. [24] found that the strength reached the maximum when the Si/Al ratio was equal to 1.90.

When the Si/Al ratio is less than 1.90, increasing the Si/Al molar ratio is conducive to increasing the number of precursor materials for the dissolution process of metakaolin. This can explain the experimental phenomena from the perspective of molecular structure. In the geopolymer reaction system, the Al(OH)_4_^−^ monomer cannot exist alone for a long period, and the polycondensation reaction with Si(OH)_4_ will occur quickly. However, the rate of polycondensation of Si(OH)_4_ monomers to form silicate oligomers is relatively slow. In a low Si/Al ratio system, the free Si(OH)_4_ and Al(OH)_4_^−^ monomers bond to each other and polycondensate in the form of hydrated ions to form a long chain (-Si-O-Al-O-) PS type geopolymer (Si/Al = 1) with low oligomeric dimer morphology and structural strength. When the Si/Al ratio is high, the continuous increase of Si content will enhance the content of dissolved elemental silicon and the increase of Si(OH)_4_ monomer will promote the formation of more -Si-O-Si- bonds, forming a more stable bond structure. Generally, the higher the Si content, the more stable it is, because the chemical bond strength of -Si-O-Si- bond is stronger than that of -Si-O-Al- and -Al-O-Al-, and due to the higher the energy required to break the chemical bond. With Si/Al increasing to a certain extent, the content of elemental silicon dissolved in the system is much higher than that of aluminum, meanwhile, a part of Si(OH)_4_ will form a dimer after condensation reaction and then react with Al(OH)_4_^−^ to form a stable long chain (-Si-O-Al-O-Si-O-) PSS polymer (Si/Al = 2) or more stable long chain(-Si-O-Al-O-Si-O-Si-O-) PSDS polymer (Si/Al = 3).

**Figure 5:**
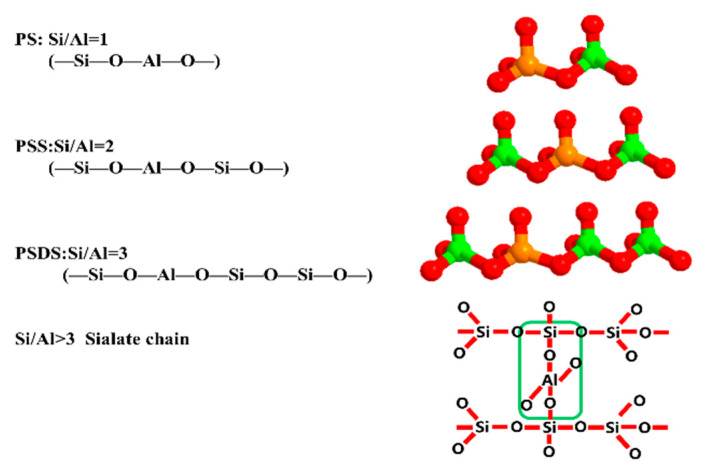
Geopolymer models with different Si/Al molar ratios.

A structural diagram is shown in Figure 5. Subsequently, the chemical system is gradually transferred from mono-silicate chains and cyclic trimers to species with larger rings and more complex polymer structures, forming a 3D polymer skeleton and improving the mechanical properties of the resulting geopolymer materials. However, it can be observed from Figure 4 that when the Si/Al ratio continues to increase to 1.95, the compressive strength does not increase significantly, but decreases to a certain extent, with the flexural strength in particular showing a sharp decline.

**Figure 6:**
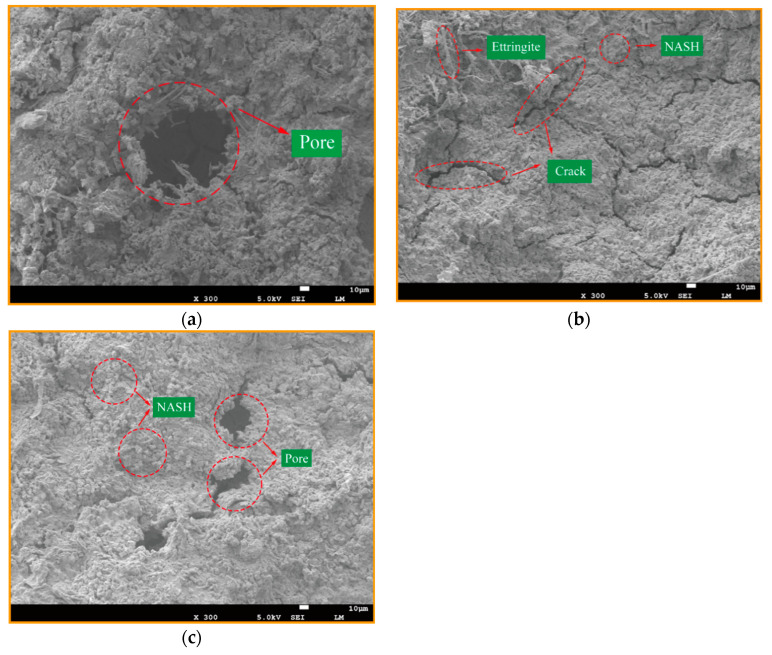
SEM images of geopolymers synthesized at various Si/Al ratios, (**a**) Si/Al = 1.7, (**b**) Si/Al = 1.9, (**c**) Si/Al = 1.9.

The reason may be that the dissolution of the reaction process reaches a saturated state in a certain alkaline environment. At the same moment, the high Si/Al ratio will inhibit the dissolution and release of the precursor in the alkaline environment, which will lead to a large number of unreacted metakaolin particles in geopolymer products. The agglomeration phenomenon is not conducive to the geopolymer reaction, which will affect the final strength of the geopolymer products. This can be confirmed by electron microscope, as shown in Figure 6, which shows more unreacted MK particles. It may be that the dissolution of the raw aluminosilicates in the reaction process reaches a saturation state under certain alkaline environments. At the same moment, the high Si/Al ratio will inhibit the dissolution and release of silicon oxide tetrahedron and aluminum oxide tetrahedron monomers of the precursor in an alkaline environment, resulting in a large number of unresponsive metakaolin particles remaining and agglomeration phenomenon occurring. This is not conducive to the synthesis reaction, which will affect the ultimate strength of geopolymer products [25]. Unreacted MK particles were discovered using an electron microscope.

Figure 6 gives the SEM images of GP pastes cured at 60 °C for 6 h, 25 °C ambient for 14 days. As the micrographs (Figure 6a) show, when the Si/Al ratio is equal to 1.70, various holes and fissures can be seen, containing a small amount crystalline components of zeolitic nuclei and sodalite phases, and most of these nuclei are not dispersed in the geopolymer binders, leading to weak geopolymerization and a mesoporous formation. Figure 6b, at the ratio of 1.9, reveals that the fewer the holes, the denser the structure, and the more homogeneous the geopolymer gel formed. Combined with the compressive strengths at various Si/Al ratios (Figure 4), it can be concluded that the formation of geopolymer gel NASH is the main factor influencing the strength of geopolymer, rather than the zeolites or silicate derivatives.

**Figure 7:**
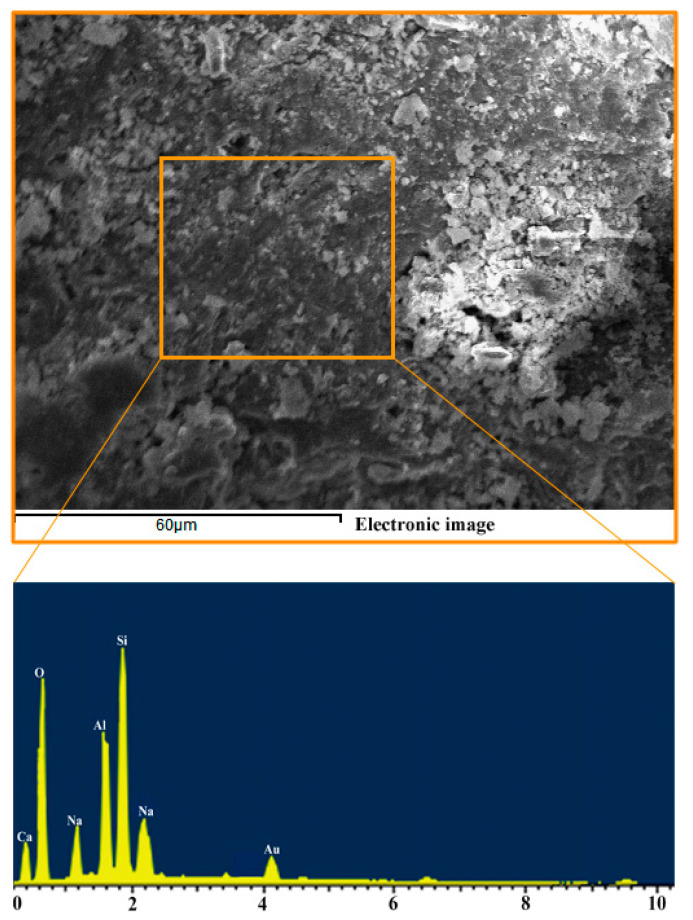
EDS spectrum of Si/Al = 1.9.

In the EDS spectrum in Figure 7, it can be seen that the content of silicon is the highest, but the content of silicon in metakaolin is lower than oxygen, which is due to the use of silicon powder as a Si/Al ratio regulator. The increase in sodium content occurs due to the use of an alkaline activator. From a quantitative point of view, we can roughly infer that Si/Al is equal to 1.90 and Na/Al is equal to 0.83 from the content of each element in EDS spectrum, which is consistent with the molar ratio used in the experiment; and the actual composition can be calculated according to the contents of SiO_2_ and Al_2_O_3_ in Table 1 and Table 3 and the silica fume.

### 3.2. The Effect of Various Na/Al Molar Ratios on a Geopolymer

**Figure 8:**
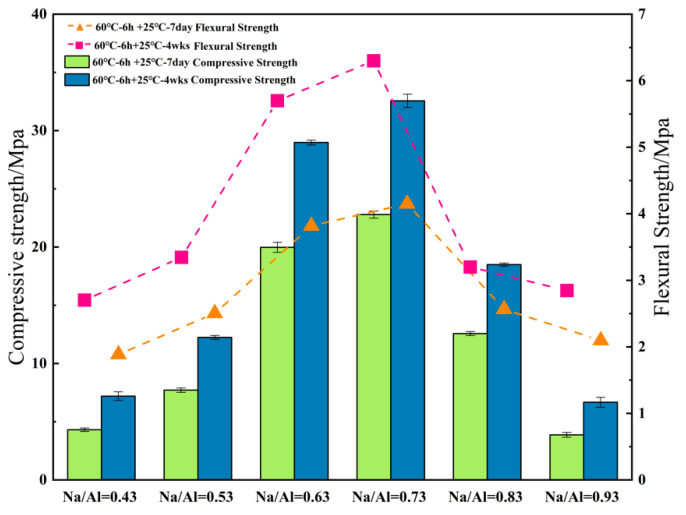
Compressive strength and flexural strength of geopolymer samples with various Na/Al ratios.

Figure 8 shows the compressive and flexural strength of geopolymers with various Na/Al ratios at various ages and it can be seen that the compressive strength of MK-geopolymer first increases then decreases with the increase of Na/Al ratio. When the Na/Al ratio is 0.73, the compressive strength and flexural strength of MK-geopolymer achieve peaks of 32.56 MPa and 6.3 MPa, and their compressive strength and flexural strength are 4.52 times and 2.32 times of those of Na/Al equal to 0.43 sample materials, respectively, after 28 curing days. However, when the Na/Al ratio exceeds 0.73, the compressive strength and flexural strength drop sharply, the compressive strength decreases to 18.49 MPa, and the flexural strength decreases to 3.2 MPa. This indicates that the Na/Al molar ratio has a considerable enhancing effect on the mechanical strength of MK-GP when the Na/Al is in a suitable range.

**Figure 9:**
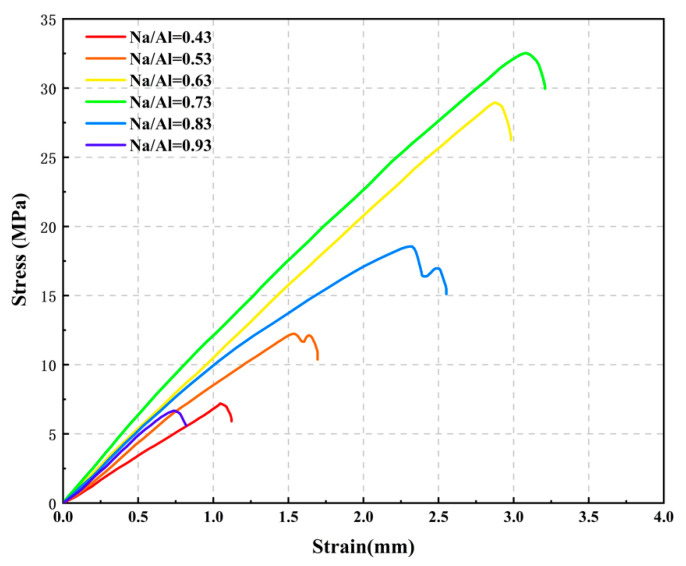
Stress–strain curves of MK-geopolymer with various Na/Al ratios at 28 days of curing.

Figure 9 shows stress–strain curves of sample with various Na/Al molar ratios. All curves manifest linear elasticity and then brittle failure, except the Na/Al equal to 0.83 and 0.53. The reason may be that when the Na/Al is relatively high, excessive sodium ions will increase the alkaline environment in the solution. The high alkali solution can promote the coagulation and hardening of the polymer. However, the excessive rate of coagulation will lead to premature crystallization precipitation of aluminosilicate gel, retained in the form of particles in the system, and the raw MK-materials cannot be fully dissolved, thus reducing the performance of the geopolymer materials. In addition, when the Na/Al ratio is high, too much alkali will also react with CO_2_ and carbonize in the air, reducing the compressive strength.

**Figure 10:**
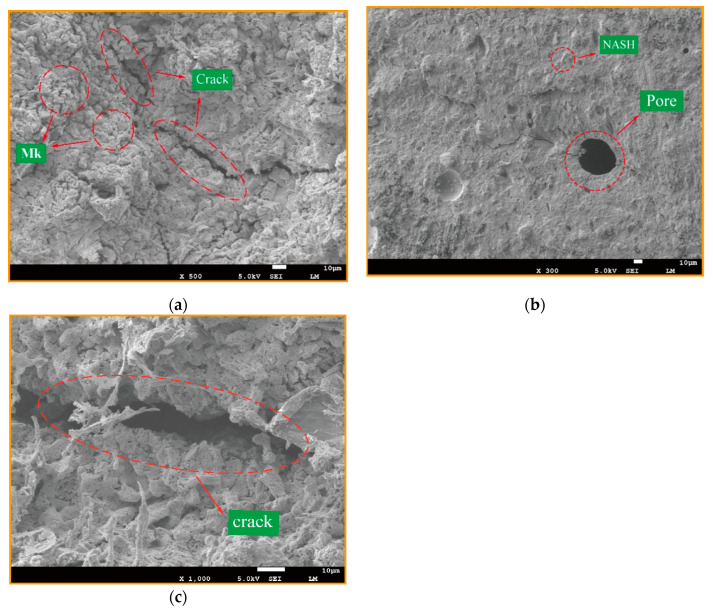
SEM micrographs of geopolymers synthesized at Na/Al, with fixing the Si/Al molar ratio, (**a**) Na/Al = 0.43, (**b**) Na/Al = 0.73, (**c**) Na/Al = 0.93.

As shown in Figure 10a, at the Na/Al ratio of 0.43, the micrograph shows flaky and colloidal particle clusters. These granular particles may be unreacted RM powder or silicon powder. The reason for the existence of granular powder is that the alkali concentration strength is too low, so that the alkali activator is not sufficient to dissolve all the precursor materials. In addition, it is noted that the microstructure is relatively loose and the pore structure is large at Na/Al = 0.43, because the geopolymer gel reaction is not sufficient to form a dense three-dimensional network structure. Compared with the Na/Al = 0.73 in Figure 10b, the microstructure of Na/Al = 0.73 is denser, showing a large number of NASH geopolymer gels. Nevertheless, when the alkali content exceeds a certain range, it is not conducive to the geopolymer reaction. As shown in Figure 10c, when the Na/Al ratio is 0.93, it shows a flaky and needle morphology. This is because the increasing Na/Al will increase the alkali content in the system, resulting in a very high alkalinity environment, which destroys the formation of geopolymer gels and reduces the structural strength.

### 3.3. Investigation into the Changes to Water in Geopolymerization

**Figure 11:**
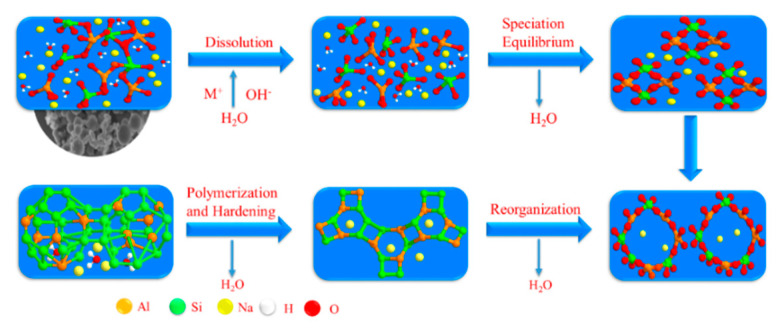
Structural model of geopolymerization.

A simplified reaction process of a geopolymer model is shown in the Figure 11. First, the raw aluminosilicates were dissolved under the attack of the alkali activator and the monomer is released. Then, the silica-aluminum monomer reacted with the alkaline solution to generate a series of products, which continuously accumulated and produced oligomeric polymers. With the reaction processing, the oligomeric state was transformed to high-polymer state, and finally the geopolymer products were formed.

From Figure 11, the whole reaction seemed to be a linear process, but the actual reaction process was almost synchronous in time and involved dissolution, reconstruction, and polycondensation simultaneously. Throughout the reaction process, water molecules were essential components. In the initial hydrolyzing stage of the reaction, the water molecules acted as reaction media as a solvent for hydroxyl ions, promoting the dissolution of silicoaluminate. However, excessive water would dilute the concentration of hydroxide ions, thereby reducing the reaction rate. In the latter stage of the second reaction, water molecules were treated as reaction products and released.

Some scholars thought that the number of water molecules involved in the dissolution process was approximately equal to the number of water molecules released in the polymerization process and that the water molecules maintained the phase equilibrium in the whole process. As shown in Figure 11, a change of water molecules in the reaction process can be observed. In order to explore the alteration of bound water and free water in the process of geopolymer reaction, the samples with Na/Al = 0.83, Si/Al = 1.65, 1.75, 1.85, and 1.95 were cured in the oven for 6 h. During this period, one group was sealed with plastic film and the other was not sealed, and then the film was removed during demolding. The mass change of the samples was monitored with ambient temperature curing for 14 days.

**Figure 12:**
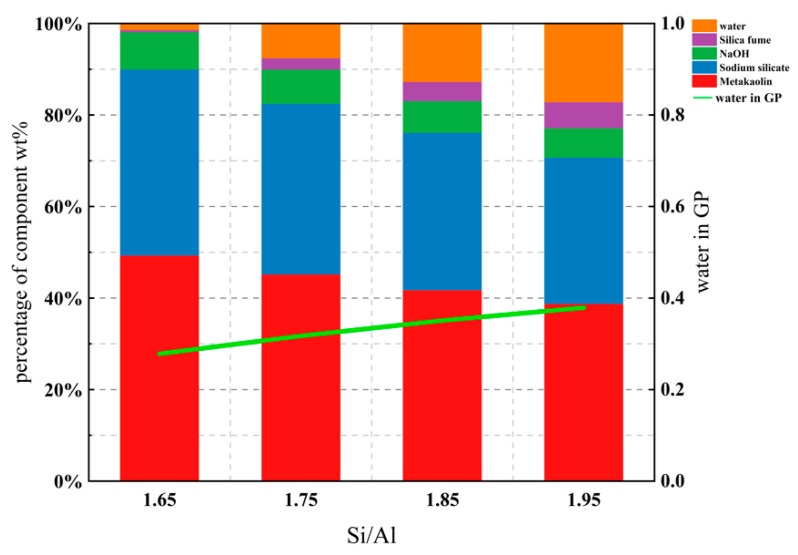
Initial geopolymer constituents and water.

The initial water content of the geopolymer raw materials and each component is shown in Figure 12, and calculated according to the residual water Equation (2).

**Figure 13:**
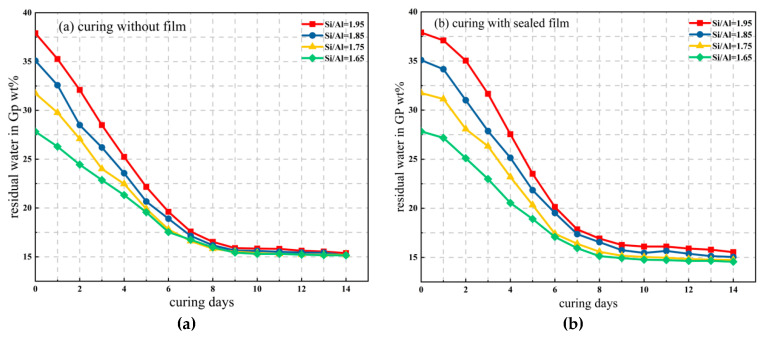
Water remaining in samples with various Si/Al ratios during curing in sealed and unsealed molds for 6 h and aging for 14 days in ambient environment, (**a**) curing without film, (**b**) curing with sealed film.

In first curing day of 6 h, it was shown that before the film was removed, the water evaporation in the sealed sample was relatively slow, which was reflected in the slope of the graph being relatively smooth, while the water evaporation in the unsealed sample was greater, showing a steep straight line of development in Figure 13. Probably, the plastic film prohibits rapid water evaporation, resulting in a lower initial water evaporation. After curing for 6 h, the film was removed and demolded. It was observed that both sealed and unsealed samples had similar outcomes, that is, a large amount of water evaporation occurred on the first 10 days or so, and the mass changed significantly. During this time, the evaporated water was mainly free water. After 10 days, the mass loss of Si/Al = 1.65 and Si/Al = 1.75 of water tended to be stable and the mass remained basically unchanged, showing a smooth linear line segment. At this time, non-evaporated water mainly existed in the geopolymer, which played a vital role in the structural properties of the geopolymer.

In addition, Figure 13 shows that the difference between the sealed and unsealed samples was large only in the early stage of water evaporation, while the diversity was relatively small with the remaining amount of non-evaporated water after 10 days in the final stage, presenting a basically similar state; while the mass fluctuated within a small range of 1% after 14 days. Moreover, no matter how much water was contained in the initial geopolymer raw material, the water content in all samples after 16–20 days of curing was almost the same, and the bound water content of the final geopolymer was maintained at about 15%; it could also be seen that the two curves of Si/Al = 1.65 and Si/Al = 1.75 were almost overlapping after 10 days.

This phenomenon was consistent with the theory proposed by previous scholars. That is, water molecules remained unchanged during the whole process of geopolymer reaction, the water molecules consumed in the dissolution process were equal to the water molecules released in the polymerization process, and the water molecules maintained a dynamic phase equilibrium during the whole reaction process.

According to the mass calculation of free water and non-evaporated water in the experimental process, the content of bound water and non-bound water in the process of geopolymer reaction can be roughly calculated. The existing forms of water molecules in the geopolymer paste were mainly as follows:

(1) About 60% of the water was free water, also known as pore water, which mainly existed between the gel particles and pore structures of the geopolymer, while the pore diameter was generally several hundred nanometers. It was basically not affected by the physical interaction, so it could participate in the reaction of the hydration process.
(2) Another part of about 35% was combined water, also called the interlayer water, its binding force relied on capillary tension and strong hydrogen bonding, hydrating with C-A-S-H and adhering to the pore surface. The pore diameter was at a nanometers scale. This part of bound water had a significant influence on the shrinkage of the microscopic pore structure and would combine with active silicon and aluminum cations to form hydration ions.
(3) The remaining part of 5% was structural water [26], which was an integral part of the chemical structure of the hydration structural products of geopolymers. They existed in the form of hydroxyl groups in the geopolymer products, with a strong chemical bond binding ability. Destroying their chemical bonds would consume a lot of energy and also change the molecular structure and morphology, so the structure water of this part was the most stable.

## 4. Discussion and Conclusions

This article used a sodium silicate solution and sodium hydroxide as an alkali activator, with silicon powder as a regulator, and metakaolin as the raw material for a geopolymer. The influence of various Si/Al and Na/Al ratios on the mechanical properties of the sample was evaluated by fixing Na/Al and Si/Al molar ratios, respectively; with explanation using SEM, EDS, and other macroscopic characterization methods. The variation of free water and bound water during the geopolymerization reaction was also discussed in this article. Based on the results of the study and characterization, the following conclusions could be drawn:

(1) The mechanical properties of the geopolymer attained a peak value of 21.3 MPa when Si/Al was equal to 1.90. Under various Si/Al ratios, the macro-strength of a geopolymer mainly relies on the formation of NASH gel rather than the zeolites or silicate derivatives.
(2) Increasing the alkalinity within a suitable range in the polymer reaction system was beneficial to the dissolution rate of the MK and geopolymerization. Whereas, a ultra-high Na/Al molar ratio caused a high alkali environment, it would enhance the crystallization and inhibit the polyreaction, resulting in significantly worse geopolymer performance.
(3) No matter how high the initial water content in the geopolymer, the discrepancy of water evaporation was only large in the early stages. The water content of all samples was almost the same after 16–20 days curing, and the bound water content in the final geopolymer was near to 15%.
(4) On account of the influence of various external factors and raw aluminosilicate material activity, the optimal mixture ratio of geopolymer may vary. It was necessary to explore the mixing ratio to achieve an optimal performance.

This paper studied the influence of Si, Al, and Na, the most important elements in MK-Geopolymer, on the performance of the geopolymer and explained the reasons for the influence on geopolymer performance from the perspective of chemical bond formation, making up for the deficiency in micro-mechanism research. Geopolymer, as an environmentally friendly material, is the most promising material for the future. The frost resistance, refractory performance, shrinkage, and material informatics of geopolymers are worthy of further research and exploration.

## Figures and Tables

**Table 1:** Compositions of metakaolin.

Compositions	SiO_2_	Al_2_O_3_	TiO_2_	Fe_2_O_3_	CaO	MgO	Na_2_O	Others
w.t.(%)	53	39	2.71	1.76	0.17	0.9	0.3	2.14

**Table 2:** Physical properties of metakaolin.

Fineness	Activity Index	Specific Surface Area (kg/m^2^)	LOI
1250	≥110	437	1.62

**Table 3:** Mass composition of sodium silicate.

Sodium Silicate	Na_2_O (w.t.%)	SiO_2_ (w.t.%)	H_2_O (w.t.%)	Be (°C)	Modulus	Transparency (%)
Index values	8.5	26.5	65	40	3.2	≥84

Note: The modulus of sodium silicate is the molecular ratio of silica and alkali metal oxides in sodium silicate, modulus = n (SiO_2_)/N (Na_2_O); Baume degree is used to characterize the concentration of sodium silicate.

**Table 4:** The Si/Al mix design of the paste (kg/m^3^).

GP Sample	Metakaolin	Sodium Silicate	Silica Fume	NaOH	Si/Al (Molar Ratio)	Na/Al (Molar Ratio)	Water in GP (w.t.%)
MKSiAl1.65	1050	864	9.45	173.92	1.65	0.83	0.278
MKSiAl1.7	1050	864	33.54	173.92	1.70	0.83	0.298
MKSiAl1.75	1050	864	57.62	173.92	1.75	0.83	0.317
MKSiAl1.8	1050	864	81.72	173.92	1.80	0.83	0.334
MKSiAl1.85	1050	864	105.80	173.92	1.85	0.83	0.350
MKSiAl1.9	1050	864	129.89	173.92	1.90	0.83	0.365
MKSiAl1.95	1050	864	153.98	173.92	1.95	0.83	0.379

Note: MKSiAl1.65 means Si/Al = 1.65.

**Table 5:** The Na/Al mix design of the paste (kg/m^3^).

GP Sample	Metakaolin	Sodium Silicate	Silica Fume	NaOH	Si/Al (Molar Ratio)	Na/Al (Molar Ratio)
MKNaAl0.43	1050	864	104.81	43.34	1.85	0.43
MKNaAl0.43	1050	864	104.81	75.45	1.85	0.53
MKNaAl0.43	1050	864	104.81	107.56	1.85	0.63
MKNaAl0.43	1050	864	104.81	139.64	1.85	0.73
MKNaAl0.43	1050	864	104.81	173.92	1.85	0.83
MKNaAl0.43	1050	864	104.81	203.92	1.85	0.93

Note: MKNaAl0.43 means Na/Al = 0.43.

## Data Availability

No new data were created or analyzed in this study. Data sharing is not applicable to this article.
